# All-Inside Arthroscopic and Open Techniques of the Modified Broström Procedure for the Treatment of Lateral Ankle Instability: Comparison of the Times to Return to Play

**DOI:** 10.3390/medicina60060921

**Published:** 2024-06-01

**Authors:** Sang Heon Lee, Sung Hwan Kim, Sung Bum Park, Seong Rok Oh, Seung Jin Choi, Young Koo Lee

**Affiliations:** 1 Department of Orthopaedic Surgery, Soonchunhyang University Hospital Bucheon, 170, Jomaru-ro, Wonmi-gu, Bucheon-si 14584, Republic of Korea; worldking70@naver.com (S.H.L.); shk9528@naver.com (S.H.K.); kokodd3@naver.com (S.J.C.); 2 Department of Orthopaedic Surgery, Bonebridge Hospital, 214, Dogok-ro, Gangnam-gu, Seoul 06272, Republic of Korea; anybabo@daum.net; 3 Department of Orthopaedic Surgery, Gurosamsung Orthopedic Surgery Clinic, 204, Gyeongin-ro, Guro-gu, Seoul 04323, Republic of Korea; ohseongrock@gmail.com

**Keywords:** ankle instability, Brostrom operation, rehabilitation

## Abstract

*Background and Objectives*: Lateral ankle injuries are commonly encountered injuries, and the open modified Broström operation (OMBO) is the primary treatment option. Recently, an arthroscopic modification of the Broström operation (AMBO) was developed; many studies have shown that there are no significant differences in clinical and radiological outcomes between the two surgical methods. However, no studies have been conducted comparing the two surgical methods in terms of return to play (RTP) time. This study assesses the time to RTP and the functional clinical outcomes. *Materials and Methods*: Sixty patients were enrolled from January 2012 to July 2014. They were segregated into two cohorts: the AMBO group comprised 30 patients, while the OMBO group comprised another 30 patients. Each participant underwent standardized treatment and rehabilitation regimens and RTP time was measured using seven questions that explored the times to return of painless walking, running, jumping, squatting, climbing stairs, and rising up on the heels and toes. We compared the time intervals from the onset of instability to the date of surgery. Clinical outcomes were evaluated before the surgery, 6 weeks after surgery, and 6 months after surgery. The assessments included the American Orthopedic Foot & Ankle Society (AOFAS) ankle–hindfoot score, the pain visual analog scale (VAS) score, subjective satisfaction with rehabilitation, and activity level. *Results*: In terms of RTP, AMBO was associated with a shorter interval to walking without pain (7.07 ± 2.96 weeks) relative to OMBO (11.03 ± 8.58 weeks). No disparities were observed in the time to return to play (RTP) between OMBO and AMBO. While there were no discrepancies in the 6-month postoperative AOFAS or VAS scores, the 6-week postoperative VAS score was notably lower in the AMBO group compared to the OMBO group. AMBO provided a faster RTP in terms of two of the seven questions in a group exhibiting high-level physical activity. The rate of subjective satisfaction with rehabilitation was higher for AMBO than for OMBO. *Conclusions*: Aside from walking, the duration to return to play and the clinical outcomes were similar between AMBO and OMBO treatments for lateral ankle instability. AMBO is a good treatment option and should be carefully considered for athletes with lateral ankle instability. AMBO demonstrated positive outcomes in a group with higher activity levels compared to others, particularly in terms of time to RTP, subjective satisfaction, and postoperative pain.

## 1. Introduction

Lateral ankle sprains are common sports injuries characterized by damage to the lateral ankle ligaments, which include the anterior talofibular ligament (ATFL), calcaneofibular ligament (CFL), and posterior talofibular ligament (PTFL). The ATFL is the weakest of these ligaments and is most susceptible to injury. In the United States, ankle sprains are prevalent, with a reported occurrence rate of 2.15 per 1000 person-years, and they contribute significantly to healthcare expenses [1,2]. The most common mechanism involves an inversion force applied to a plantarflexed foot, leading to frequent sprains of these ligaments [3]. After an inversion injury, there is a high risk of ATFL rupture [4]. While isolated ATFL ruptures are common, injuries that are more significant often involve both the ATFL and CFL, whereas stand-alone CFL ruptures are uncommon and rarely documented [5].

Given the prevalence and impact of these injuries, a key concern for patients and healthcare providers is the timing of return to play (RTP). A common and pressing question from patients is “When can I (or my player) return to sports?” [6]. This highlights the need for effective treatment approaches that facilitate rapid RTP. Therefore, we often deliberate on the best treatment strategy to ensure swift recovery for patients with ankle sprains.

The initial approach to treating acute lateral ligament injuries typically involves conservative methods such as rest, ice application, compression, limb elevation, and functional rehabilitation [7]. If patients fail to respond to rehabilitation, there are various surgical alternatives. These are generally categorized into non-anatomical and anatomical methods [8]. Nonanatomic approaches do not recreate the normal anatomic insertion points of ligaments; instead, such procedures use local grafts (e.g., a peroneus brevis tendon autograft) to reconstruct the attenuated ligament. Initially, non-anatomical reconstructions were generally associated with favorable short-term results. However, some patients continued to suffer from ongoing pain, stiffness, wound complications, and impaired function of the ankle and subtalar joints [9]. As a result, the prevalence of these non-anatomical techniques has diminished over time. In 1966, Broström developed an anatomical surgical technique that repairs the damaged ligament directly by utilizing the remnants of the ATFL, thereby preventing muscle imbalance. This procedure was further refined in 1980 by Gould, who reinforced it with the inferior extensor retinaculum to enhance mechanical strength. Gould’s modifications aimed to overcome the limitations of earlier methods, such as procedural complexity, extended immobilization periods, and the potential for ankle degeneration. The primary benefits of the Gould modification include its straightforwardness, preservation of subtalar joint mobility, restoration of normal joint anatomy and movement, and a lower rate of intraoperative and postoperative complications compared to traditional techniques [10]. The open modified Broström operation (OMBO) is currently standard procedure for the treatment of lateral ankle injuries (LAIs). Over the past decade, interest in arthroscopic surgery has grown; this increased interest enables thorough assessment of the intra-articular pathology [11]. Several studies showed no significant differences in clinical or radiological outcomes between the arthroscopic modified Broström operation (AMBO) and the OMBO, and no differences in biomechanics [12,13].

The rehabilitation program for chronic ankle instability consists of both functional and preventative components. Throughout both phases, patients engage in multidirectional strengthening exercises, focusing on proprioceptive training, strengthening of the peroneal muscles, and sport-specific training for athletes [14]. Most patients achieve satisfactory results after rehabilitation, bracing, and physical therapy [7]. However, 32% of ankle sprain patients report chronic pain, swelling, or recurrent sprains after conservative treatment [15]. Among all sport injuries, the recurrence rate of lateral ankle sprains is notably high [16]. The sequence of events initiated by ligamentous trauma includes clinical manifestations such as recurrent sprains and episodes of “giving way”. The common etiologies of ankle post-traumatic osteoarthritis include histories of both single and recurrent ankle sprains [17]. Furthermore, such injuries may become exacerbated considering the increased susceptibility to long-term symptoms, triggering chronic ankle instability [18]. Many authors have identified various impairments associated with chronic ankle instability, including a reduced range of motion (ROM), loss of strength, impaired postural control, and altered movement patterns during functional activities compared with individuals who have no history of lateral ankle sprain [19].

Despite the effectiveness of conservative treatments, surgical intervention is often preferred for athletes and other patients who require rapid recovery, as dysfunctions can persist in 40% of patients for up to six months post-injury [20]. The minimally invasive nature of AMBO suggests it might reduce recovery time and morbidity, potentially leading to a faster RTP.

We hypothesized that a rapid RTP could be achieved after arthroscopic procedures, which are minimally invasive and may thus reduce the recovery time and morbidity. The purpose of this study was to compare the patient’s satisfaction of surgery and the RTP time of the AMBO and OMBO for LAI.

## 2. Materials and Methods

### 2.1. Patient Enrollment

In this retrospective study, patients treated for LAIs with MBO surgery from January 2012 to July 2014 were included. LAIs were diagnosed via (1) recurrent instability of the ankle secondary to an injury to the lateral ligament complex after failure of nonoperative management, including physical therapy, bracing, and immobilization; (2) giving way; and (3) persistent pain. The exclusion criteria were (1) previous fractures of the affected ankle, (2) prior surgery, (3) a mal-aligned foot, and/or (4) any neuromuscular disorder. Sixty patients were ultimately enrolled; 30 patients were treated with AMBO, while an another 30 were treated via OMBO procedures. Patients were divided into two groups according to activity level: recreational athletes, and sedentary individuals [21]. Preoperative demographic data showed no differences between the two groups (age, sex, activity level, or “onset op date”). The mean age was 34.9 years. Males made up 55.0% of the total sample, while females made up 45.0%, with no significant difference (*p* = 0.194). Recreational athletes accounted for 51.7% of the total. The mean onset operation date was 10.70 days overall, with 10.53 days for the open surgery group and 10.87 days for the arthroscopy group (*p* = 0.874) (Table 1).

### 2.2. Clinical Evaluations

Patients underwent preoperative evaluation, followed by assessments at 6 weeks and 6 months postoperatively. All patients underwent clinical evaluations utilizing the American Orthopedic Foot & Ankle Society (AOFAS) ankle–hindfoot scores and a visual analog scale (VAS) for pain assessment. Any concomitant injuries, supplementary procedures, or complications were also documented. Cosmetic satisfaction was assessed as excellent, good, fair, or poor. All surveys were directly completed by the patients, who were then evaluated by orthopedic specialists (Y.K.L.) in outpatient settings.

### 2.3. Activity Levels and RTPs

We aimed to assess the RTPs across our patients by posing seven questions targeting various ankle movements: walking, running, jumping, squatting, stair climbing, and rising up on heels and toes without experiencing discomfort. Patients recorded the number of weeks post-surgery at which they were able to perform relevant activities. For activities like walking and running, the recorded number indicated the weeks during which they could engage in the activity for 10 min. For activities such as jumping, squatting, stair climbing, and rising up on heels and toes, the recorded number indicated the weeks at which they began the activity. We provided education on these activities and rehabilitation both before and after surgery, and consistently monitored and verified patient responses during outpatient visits to minimize errors.

## 3. Surgical Technique

A sole surgeon (Y.K.L.) conducted all the procedures. Surgeries were carried out under either spinal anesthesia or general anesthesia combined with a thigh tourniquet. Patients were positioned supine with a bolster beneath the corresponding buttock.

### 3.1. Open MBO

In open MBO, the procedure involved ATFL imbrication with reinforcement of the inferior extensor retinaculum. A 5 cm curved incision was created just anterior to the fibular border, situated between the superficial peroneal and sural nerves. The torn or excessive segment of the ATFL was partially removed, followed by the repair of the remaining tissue using 2-0 Ethibond while the ankle was maintained in a neutral position. Subsequently, the repair involved suturing the extensor retinaculum to the periosteum along the fibula, employing the “pants-over-the-vest” technique as described by Gould et al. [22]. Upon confirming stability, the incision was sutured closed [23].

### 3.2. All-Inside Arthroscopic MBO

Prior to anchor insertion, the accessory fiber of the distal tibiofibular ligament underwent shaving until it reached its fibular insertion point. Right below the anterior tibiofibular ligament the synovial tissue and periosteum were removed, exposing a bleeding bone surface using a motorized burr. A perpendicular drill hole was then created on the anterior surface of the fibula, through which the anchor was inserted via the anterolateral portal.

**Figure 1:**
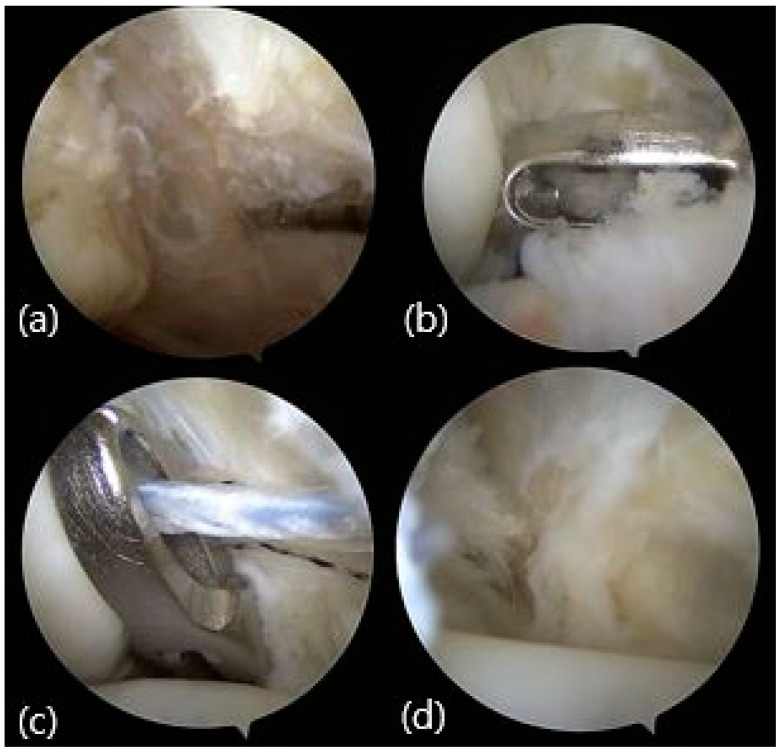
Arthroscopic pictures of all-inside AMBO in left ankle. (**a**) Ruptured ATFL of the left ankle shown arthroscopically. (**b**) Drilling to insert the suture anchor at the front surface of the fibula. (**c**) Penetrator was used to grasp each suture. (**d**) The sutures were subsequently drawn taut, while the foot was positioned in eversion and dorsiflexion.

An absorbable Bio-Suture Tak anchor was prepared with two sutures: a FiberWire and a TigerWire (Arthrex, Naples, FL, USA). An additional portal was established anteriorly and inferiorly within the sinus tarsi area. Furthermore, a portal was crafted laterally beyond the anterior fibula. Employing a penetrator, which grasped a single extremity of each suture, the threads were guided intra-articularly from the anterolateral portal to the newly established lateral portal. Subsequently, the other end of each suture was pulled out through the far-lateral portal using the penetrator. A suture retriever was inserted through the far-lateral portal and then maneuvered to the accessory anteroinferior portal. The retriever subcutaneously retrieved the sutures, and the sutures were subsequently drawn taut, while the foot was positioned in eversion and dorsiflexion [24] (Figure 1).

### 3.3. Rehabilitation Protocol

Each patient was provided with a well-padded posterior splint, maintaining slight dorsiflexion of the foot, and was instructed not to bear weight until the 2-week follow-up. For the subsequent 2 weeks, they were put in a short-leg walking cast and given protection; after this period, progressive weight bearing was permitted. In weeks 4 to 6, patients transitioned to a half-removed cast or splint and initiated gentle active-assisted range of motion (ROM) exercises for the ankle and peroneal strengthening. Around 8 weeks after surgery, patients were advised to start straight running and functional activities. By week 12, they began cutting and sports-specific drills. Following this phase, all patients were cleared to resume their sports activities without any restrictions.

### 3.4. Statistical Analysis

Demographic characteristics are summarized as frequencies (percentages) for qualitative variables and means ± standard deviations (SDs) for quantitative variables. Comparisons between the groups were made using χ^2^ tests for qualitative variables and the independent two-sample *t*-test for quantitative variables. SPSS Statistics version 20.0 (IBM Corp., Armonk, NY, USA) and SAS version 9.4 (SAS Institute, Cary, NC, USA) were used to perform all statistical analyses. A *p*-value < 0.05 was considered statistically significant. Efficacy was assessed using a linear mixed model (LMM) with unstructured covariance. The VAS and AOFAS scores served as dependent variables. The operation method, time of visit, and interaction between operation and time were treated as fixed effects, while the patients and error were considered random effects. To model the covariance structure within patients, compound symmetry was utilized. No imputations for missing data were performed. All comparisons were made at the time of visits, and tests for the group effects were conducted with a two-sided significance level of 0.05. Ninety-five percent confidence intervals (CIs) for the differences between operation methods were calculated based on AMBO values minus OMBO values.

## 4. Results

### 4.1. Patient Characteristics

Of the 60 patients in the study cohort, 30 underwent OMBO and 30 underwent AMBO. The characteristics of the cohort are summarized in Table 1. We investigated the patient’s follow-up only for up to 6 months. No differences in demographic characteristics were found between the two groups.

### 4.2. RTPs by the Surgical Method

We retrospectively investigated times to RTP postoperatively and recorded the weeks when RTPs began. In Table 2, the AMBO group shows faster RTPs than the OMBO group, but only walking normally over 10 min showed statistical significance (*p* = 0.02). The other RTPs did not differ significantly (*p* = 0.953, 0.687, 0.323, 0.565, 0.089, and 0.133).

### 4.3. RTP Differences by Activity Level

We also asked patients about their usual activity level and categorized patients by activity. There were no professional or amateur athletes in enrolled patients. We therefore compared RTPs in recreational athletes and the sedentary group. The group reporting high-level physical activity (recreational athletes) experienced relatively short RTPs than the low-level physical activity group (sedentary), but there was no significant difference in the OMBO group’s RTPs (*p* = 0.238, 0.946, 0.466, 0.371, 0.732, 0.693, and 0.88). In the AMBO group, squatting (*p* = 0.024) and rising up on the heels of the operated leg (*p* = 0.004) were significantly different according to the activity level, but no significant difference was found among the other RTPs (*p* = 0.469, 0.964, 0.267, 0.172, and 0.238) (Table 3).

Recreational athletes who underwent AMBO exhibited significantly shorter RTPs in terms of walking normally over 10 min (*p* = 0.012) and rising up on the heels without pain (*p* = 0.003), but they showed no differences among the other RTPs (*p* = 0.987, 0.656, 0.196, 0.37, and 0.064). The sedentary group exhibited no difference in RTP duration according to the operative method (*p* = 0.109, 0.927, 0.779, 0.572, 0.97, 0.965, and 0.557) (Table 3).

### 4.4. Clinical Outcomes and Subjective Satisfaction

**Figure 2:**
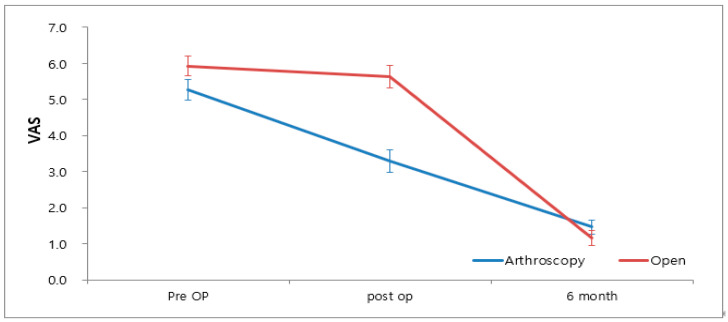
VAS pain score according to the operative method.

The VAS scores of the AMBO and OMBO groups were lower at 6 weeks and 6 months postoperatively, compared with those preoperatively (*p* < 0.001). No significant difference was observed between the two groups preoperatively (*p* = 0.102) or at 6 months postoperatively (*p* = 0.293). However, at the 6-week postoperative mark, the AMBO group had a significantly lower score compared to the OMBO group (*p* < 0.001) (Figure 2). According to the mixed model result, the two groups showed significant differences with time (*p* < 0.001).

**Figure 3:**
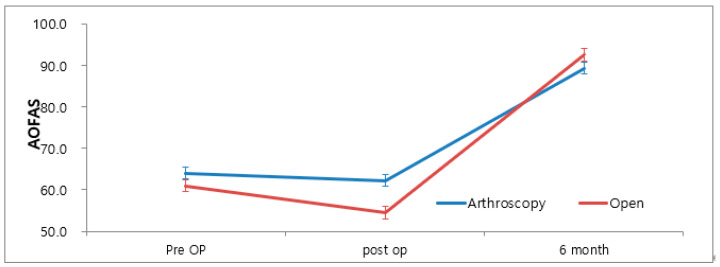
AOFAS score according to the operative method.

The AOFAS score of the AMBO and OMBO groups showed a slight decrease postoperatively and improved at the postoperative 6-month follow-up (*p* < 0.001). There was no significant difference between the two groups preoperatively (*p* = 0.422) or 6 months postoperatively (*p* = 0.349), but the 6-week postoperative AOFAS score of the AMBO group was significantly higher (*p* < 0.001) (Figure 3). According to the mixed-model test, the two groups showed significant differences with time (*p* < 0.001).

A higher percentage of patients in the AMBO group (80%) reported excellent subjective satisfaction compared to the OMBO group (27.6%), although the difference between the two groups was not statistically significant (Table 4).

## 5. Discussion

The key findings in this study were that postoperative pain and functional limitations were significantly lower in the AMBO group compared to the OMBO group. While there was no significant difference in the overall time to return to play (RTP), some parameters for a group engaging in high physical activity showed significant differences favoring the AMBO group. Cosmetic satisfaction did not significantly differ between the groups, but the AMBO group reported better satisfaction. Postoperative pain, as measured by the Visual Analog Scale (VAS), was significantly lower in the AMBO group at 6 weeks post-surgery, suggesting that these patients experienced less pain in the early postoperative period, potentially facilitating faster rehabilitation. Functional outcomes, evaluated using the American Orthopedic Foot & Ankle Society (AOFAS) score, showed no significant difference between the two groups preoperatively or at 6 months postoperatively; however, at 6 weeks postoperatively, the AMBO group had significantly better functional scores. This early improvement in function could be crucial for patients aiming for a quicker return to their daily activities and sports. Although there was no significant difference in the overall RTP time between the groups, the time to return to painless walking was significantly shorter in the AMBO group (7.07 ± 2.96 weeks) compared to the OMBO group (11.03 ± 8.58 weeks), indicating that AMBO may benefit patients prioritizing a swift return to basic physical activities. Additionally, 80% of the patients in the AMBO group rated their satisfaction with the rehabilitation process as excellent, compared to 27.6% in the OMBO group, reflecting the overall better early postoperative outcomes observed in the AMBO group.

Arthroscopy has been used by orthopedic surgeons for decades. Currently, almost all joints can be operated with arthroscopy, and there has been a dramatic change in the approach of diagnosis and treatment of various joint problems. In knee and shoulder problems, arthroscopic surgery is usually decided. Most anterior and posterior cruciate ligament injuries of the knee require an arthroscopic procedure and open procedures are rare. Arthroscopic and mini-open repair of shoulder rotator cuff tears did not differ in terms of surgery time, functional outcome score, VAS pain score, or ROM at the end of follow-up [25]. Recent advancements in arthroscopic techniques have enabled the early diagnosis of intra-articular pathology through minimally invasive methods. Furthermore, these advancements have made it possible to perform lateral ligament repair through arthroscopy. One cadaveric study revealed no differences in degrees to failure, torque to failure, or stiffness for the repaired ligament complex between conventional open Broström repair and direct arthroscopic anatomical repair [26]. 

In 2012, Kerkhofs et al. published evidence-based clinical guidelines that covered many effective treatment options for individuals with acute lateral ankle sprain injuries [27]. In their study, exercise therapy appears to prevent recurrence in patients with lateral ankle instability over the long term (8 to 12 months), despite showing no significant effect in the medium term (6 to 9 months). However, individuals with lateral ankle ligament injuries often fail to receive or pursue ongoing appropriate treatment and patient-centered rehabilitation [28]. A recent update of the evidence-based clinical guidelines recommended that clinicians implement multifaceted, exercise-based rehabilitation programs to facilitate a faster return to sports by individuals with acute lateral ankle sprain injuries [29]. Exercise therapy should be initiated following lateral ankle sprain to maximize the recovery of joint functionality. However, whether exercise therapy should be supervised or not remains unclear due to conflicting evidence, and warrants further investigation.

When assessing an athlete’s readiness to return to sports activities, it is essential to ensure that all functional limitations resulting from the injury are addressed, cardiovascular fitness is at least equal to pre-injury levels, and there is no apprehension regarding the athlete’s health and safety from either the athlete or other members of the rehabilitation team [30]. Residual disability after an ankle joint sprain is often attributable to an inadequate rehabilitation program and/or premature RTP. The time required for RTP after lateral ligamentous ankle sprains varies depending on several factors, including the severity of the injury, the athlete’s capacity, and the availability of rehabilitation resources. In a case series involving professional athletes who underwent surgical ligament repair, the median times to RTP were 77 days for those with isolated lateral ligamentous injuries and 105 days for those with concomitant injuries [31]. Russo et al. performed OMBO on 18 athletes involved in competitive sports at different levels; all exhibited LAIs and all returned to their various sporting activities at the levels prior to surgery [32]. White et al. used OMBO to treat 42 professional athletes with confirmed acute grade III lateral ligament injuries; all ankles stabilized, and all athletes returned to sports at approximately 10 weeks [33]. Nolan et al. reported that 54% of patients returned to their pre-injury activity levels after OMBO [26]. Among the 19 athletes who did not return to their pre-injury level of activity, 7 (37%) attributed their non-return to ankle-related reasons, while 12 (63%) cited non-ankle-related reasons. The most prevalent non-ankle-related reason was a change in the level of play after graduation. The ankle-related reasons included pain (57%), residual instability (29%), and decreased ROM (14%). Lee et al. reported RTP rates of 83.3% at 4 months and 100% at 8 months after OMBO to treat chronic ankle instability in elite athletes [34]. In that study, the average times were 1.9 months to resume personal training, 2.9 months to rejoin team training, and 3.9 months to rejoin competitive play. There were no significant differences between the early and late RTP groups in terms of age, sex, body mass index (BMI), level of sport, instability grade, os subfibulare status, or preoperative functional score.

In the current study, factors that could potentially impact the time to RTP were analyzed between the early and late RTP groups. These variables included age, sex, BMI, instability grade, and os subfibulare status. Earlier studies indicate that younger athletes tend to return to their pre-injury sports level more readily after anterior cruciate ligament reconstruction [35]. Moreover, there was approximately a 1.5-fold higher likelihood for men compared to women to resume their previous level of sport or competitive sports [36]. Female sex and syndesmotic injury negatively influenced the RTP in patients who underwent surgery to treat ankle fractures. Furthermore, competitive athletes demonstrated a lower likelihood of returning to sports in comparison to recreational athletes [37]. Choon et al. reported that use of the modified Broström repair to treat elite athletes with isolated, acute, grade III ankle lateral ligament injuries allowed for predictable RTP within a mean of 10 weeks but that a concomitant deltoid or syndesmosis injury may delay return by approximately 1 week [38].

However, there is no study comparing the subjective satisfaction and RTP between AMBO and OMBO. The importance of a faster and better RTP can be considered because this ankle instability can be a carrier ending disease in athletes. The difference in RTP between the two groups aids in the identification of a better treatment modality. There was no notable distinction observed in the duration to RTP between the AMBO and OMBO groups, but subjective satisfaction data indicated that AMBO was better than OMBO, and it seems that ABMO has advantages over OMBO.

This study had some limitations. The sample size was limited. Despite conducting a power analysis for the sample size, there remains susceptibility to beta errors. Retrospective surveys were performed in this paper. There was no difference in rehabilitation protocols between the OMBO and AMBO groups. All patients followed the rehabilitation protocol of our hospital, and the OMBO and AMBO groups exhibited similar times to RTP. Because the 6-week postoperative VAS score was significantly lower in the AMBO group, rehabilitation could have been faster in that group. No professional or amateur athlete was enrolled. Thus, we could not confirm whether patients returned to various sports activities at the same levels as before surgery. Additionally, the patient population was not diverse; our results may not be generalizable. The follow-up was only 6 months, so more scheduled follow up seems to be needed after gathering more data. To address the limitations mentioned, a prospective, randomized controlled trial (RCT) design could be implemented. This design would help overcome the key limitations, such as small sample size, retrospective nature, and lack of diversity in the patient population.

## 6. Conclusions

Although it was anticipated that patients undergoing AMBO would experience a shorter RTP, no significant differences were observed between the AMBO and OMBO groups. Patients who underwent AMBO reported significantly less pain at 6 weeks post-surgery compared to those who underwent OMBO, and the significant RTP difference observed was mainly in the category of walking which showed a relatively faster recovery time. This indicates that the degree of postoperative pain may play a more crucial role in RTP time than other potential factors. There appears to be a tendency for a shorter RTP in patient groups demonstrating high-level physical activity. It suggests that further studies including patients engaging in higher levels of physical activity, such as amateur and professional athletes, could potentially reveal significant RTP differences between groups undergoing ABMO and OMBO, particularly within the timeframe where AMBO demonstrates significant differences in pain.

## Figures and Tables

**Table 1:** Baseline characteristics according to the operative method.

Variable	Total	Open	Arthroscopy	*p*-Value
	(*N* = 60)	(*N* = 30)	(*N* = 30)	
Age (years)	34.9 ± 12.8	37.43 ± 15.59	33.57 ± 11.94	0.285
Sex				0.194
Male	33 (55.0)	14 (46.7)	19 (63.3)	
Female	27 (45.0)	16 (53.3)	11 (36.7)	
Physical activity				0.796
Recreational athletes	31 (51.7)	16 (53.3)	15 (50.0)	
Sedentary	29 (48.3)	14 (46.7)	15 (50.0)	
Onset op date	10.70 ± 8.06	10.53 ± 7.87	10.87 ± 8.38	0.874

**Table 2:** Time to recovery according to the operative method (weeks).

Variable	Open	Arthroscopy	*p*-Value	Effect Size
Walking	11.03 ± 8.58	7.07 ± 2.96	0.020	0.46
Running	19.03 ± 13.24	18.87 ± 7.80	0.953	0.01
Jumping	20.40 ± 16.88	18.97 ± 9.56	0.687	0.05
Squatting	27.87 ± 26.41	19.73 ± 9.88	0.323	0.21
Climbing stairs	26.13 ± 14.73	24.20 ± 10.85	0.565	0.07
Rising up on heels without pain	23.60 ± 14.81	18.13 ± 8.97	0.089	0.27
Rising up on toes without pain	22.00 ± 14.13	17.43 ± 8.39	0.133	0.09

**Table 3:** Recovery times according to the operative method and activity level (weeks).

Variable	Recreational Athletes	Sedentary	*p*-Value	Effect Size
Open				
Walking	9.13 ± 2.06	13.21 ± 12.14	0.238	0.24
Running	18.88 ± 12.41	19.21 ± 14.60	0.946	0.00
Jumping	18.25 ± 5.70	22.86 ± 24.21	0.466	0.11
Squatting	20.75 ± 12.37	29.57 ± 36.53	0.371	0.21
Climbing stairs	25.25 ± 11.77	27.14 ± 17.95	0.732	0.07
Rising up on heels without pain	24.63 ± 11.19	22.43 ± 18.50	0.693	0.09
Rising up on toes without pain	21.63 ± 10.334	22.43 ± 17.94	0.880	0.02
Arthroscopy				
Walking	6.67 ± 2.90	7.47 ± 3.07	0.469	0.12
Running	18.93 ± 6.71	18.80 ± 9.00	0.964	0.01
Jumping	17.00 ± 9.19	20.93 ± 9.82	0.267	0.23
Squatting	15.73 ± 8.17	23.73 ± 10.05	0.024	0.39
Climbing stairs	21.47 ± 11.33	26.93 ± 9.97	0.172	0.36
Rising up on heels without pain	13.60 ± 7.06	22.67 ± 8.54	0.004	0.51
Rising up on toes without pain	15.60 ± 6.51	19.27 ± 9.80	0.238	0.22
**Variable**	**Open**	**Arthroscopy**	***p*-Value**	**Effect Size**
Recreational athletes				
Walking	9.13 ± 2.06	6.67 ± 2.90	0.012	0.16
Running	18.88 ± 12.41	18.93 ± 6.71	0.987	0.01
Jumping	18.25 ± 5.70	17.00 ± 9.19	0.656	0.08
Squatting	20.75 ± 12.37	15.73 ± 8.17	0.196	0.19
Climbing stairs	25.25 ± 11.77	21.47 ± 11.33	0.370	0.22
Rising up on heels without pain	24.63 ± 11.19	13.60 ± 7.06	0.003	0.49
Rising up on toes without pain	21.63 ± 10.334	15.60 ± 6.51	0.064	0.14
Sedentary				
Walking	13.21 ± 12.14	7.47 ± 3.07	0.109	0.18
Running	19.21 ± 14.60	18.80 ± 9.00	0.927	0.02
Jumping	22.86 ± 24.21	20.93 ± 9.82	0.779	0.07
Squatting	29.57 ± 36.53	23.73 ± 10.05	0.572	0.12
Climbing stairs	27.14 ± 17.95	26.93 ± 9.97	0.970	0.01
Rising up on heels without pain	22.43 ± 18.50	22.67 ± 8.54	0.965	0.02
Rising up on toes without pain	22.43 ± 17.94	19.27 ± 9.80	0.557	0.10

**Table 4:** Subjective satisfaction according to the operative method.

**Variable**	**Open**	**Arthroscopy**	***p*-Value**
Satisfaction			>0.999
Excellent	8 (27.6)	24 (80.0)	
Good	13 (44.8)	5 (16.7)	
Fair	9 (27.6)	1 (3.3)	

## Data Availability

The original contributions presented in the study are included in the article, further inquiries can be directed to the corresponding authors.
